# Control of *gag-pol *gene expression in the *Candida albicans *retrotransposon Tca2

**DOI:** 10.1186/1471-2199-8-94

**Published:** 2007-10-25

**Authors:** Elaine M Forbes, Siân R Nieduszynska, Fiona K Brunton, Joanne Gibson, L Anne Glover, Ian Stansfield

**Affiliations:** 1 University of Aberdeen, School of Medical Sciences, Institute of Medical Sciences, Foresterhill, Aberdeen AB25 2ZD, UK; 2 Current address : Dept. of Pediatrics, University of Arizona, College of Medicine, Tucson, AZ 85724, USA; 3 Current address : School of Medicine, King's College London, Strand, London, WC2R 2LS, UK

## Abstract

**Background:**

In the *C. albicans *retrotransposon Tca2, the *gag *and *pol *ORFs are separated by a UGA stop codon, 3' of which is a potential RNA pseudoknot. It is unclear how the Tca2 *gag *UGA codon is bypassed to allow *pol *expression. However, in other retroelements, translational readthrough of the *gag *stop codon can be directed by its flanking sequence, including a 3' pseudoknot.

**Results:**

The hypothesis was tested that in Tca2, *gag *stop codon flanking sequences direct translational readthrough and synthesis of a *gag-pol *fusion protein. Sequence from the Tca2 *gag*-UGA-*pol *junction (300 nt) was inserted between fused *lacZ *and luciferase (*luc*) genes in a *Saccharomyces cerevisiae *dual reporter construct. Although downstream of UGA, *luc *was expressed, but its expression was unaffected by inserting additional stop codons at the 3' end of *lacZ*. *Luc *expression was instead being driven by a previously unknown minor promoter activity within the *gag-pol *junction region. Evidence together indicated that junction sequence alone cannot direct UGA readthrough. Using reporter genes in *C. albicans*, the activities of this *gag-pol *junction promoter and the Tca2 long terminal repeat (LTR) promoter were compared. Of the two promoters, only the LTR promoter was induced by heat-shock, which also triggers retrotransposition. Tca2 *pol *protein, epitope-tagged in *C. albicans *to allow detection, was also heat-shock induced, indicating that *pol *proteins were expressed from a *gag*-UGA-*pol *RNA.

**Conclusion:**

This is the first demonstration that the LTR promoter directs Tca2 *pol *protein expression, and that *pol *proteins are translated from a *gag*-*pol *RNA, which thus requires a mechanism for stop codon bypass. However, in contrast to most other retroelement and viral readthrough signals, immediate *gag *UGA-flanking sequences were insufficient to direct stop readthrough in *S. cerevisiae*, indicating non-canonical mechanisms direct *gag *UGA bypass in Tca2.

## Background

Retrotransposons are mobile genetic elements that replicate via an RNA intermediate. Their replication cycles and genome organisation are similar in many respects to those of retroviruses, and retrotransposons form a cytoplasmic virus-like particle during replication. Both groups of retroelements have a genome comprising *gag *and *pol *coding sequences. *Gag *proteins make up the capsid of the viral particle while *pol *encodes a polyprotein usually made up of integrase, protease and reverse transcriptase activities. These are cleaved from the polyprotein by the action of the viral protease [1-4].

All retroelements face the common regulatory challenge of expressing *gag *proteins in a 20:1 molar ratio to the enzymic *pol *proteins from a single RNA genome [5]. Departure from this stoichiometry causes defects in retroviral and retrotransposon replication [6-9]. Various post-transcriptional regulatory strategies are employed to maintain *gag*/*pol *stoichiometry. The *Drosophila copia *retroelement uses regulated splicing to produce *gag *proteins from one RNA, and at a lower level, *gag*-*pol *protein from a separate RNA [10,11]. Many other retroelements control *gag/pol *ratios at the level of translation. In the retrovirus HIV, the *pol *reading frame is -1 with respect to the 0 *gag *frame, and a ribosomal frameshift realigns the translational reading frame to that of the downstream *pol *cistron [12]. The *S. cerevisiae *Ty1 and Ty3 retroelements use a +1 frameshift site to regulate the *gag/pol *stoichiometry [13-16].

Another group of retroelements has either the *gag*/*pol *or *pol*/*env *reading frames separated by an in-frame stop codon. This includes Tca2 from *Candida albicans*, SIRE1 from soybean, and the well-characterised retrovirus Moloney Murine Leukaemia virus (MuLV) [17-19]. In MuLV, a purine rich spacer and then an RNA pseudoknot sequence follow the gag UAG stop codon. Both these elements are required to direct a programmed translational readthrough of the stop codon with a frequency of 5% [20,21]. The stop codon is mis-decoded by an ordinary cellular tRNA [22]. The inhibitory binding of the MuLV *pol*-encoded reverse transcriptase to the translation release factor eRF1 further enhances the readthrough frequency [23].

Retroelements are not alone in employing programmed stop codon readthrough to regulate gene expression, with Sindbis virus, tobacco mosaic virus, tobacco rattle virus, and other plant viruses employing a similar strategy [24-26]. In all cases, the immediate nucleotide context of the stop codon, in particular the six 3' nucleotides, is a key (and frequently, sole) component of the readthrough signal [27,28]. For example, readthrough in tobacco mosaic virus and other plant virus is directed solely by the consensus sequence CAR-YYA immediately 3' of the stop codon [27], and in Sindbis virus, by a cytidine residue immediately 3' of the stop codon [25]. In contrast to these viral examples of readthrough, how stop codons in other retroelements are bypassed is unknown. For this reason, we elected to study Tca2, an active retrotransposon widespread in *C. albicans *strains in which *gag *and *pol *ORFs are separated by an in-frame UGA stop codon [29]. Immediately downstream of the stop codon is an 8-nucleotide purine-rich nucleotide spacer, followed by a sequence capable of forming a bulged stem pseudoknot [17]. Structurally, this arrangement is very reminiscent of the MuLV stop codon context.

In this study we therefore examined whether Tca2 *pol *expression results from readthrough of the *gag *stop codon, enhanced by the RNA pseudoknot structure downstream. Using the closely-related model yeast *S. cerevisiae *as a test-bed system, multiple lines of evidence show that local sequences flanking the Tca2 *gag *stop codon are insufficient to direct its translational readthrough. However, Tca2 promoter analysis in *C. albicans *revealed that the expression of *pol *proteins matched the induction conditions of the retroelement long terminal repeat (LTR) promoter. Pol proteins must therefore be expressed from a *gag*-*pol *RNA, and this indicates that contrary to expectation, in *C. albicans*, *pol *protein expression is mediated by a non-canonical stop codon bypass mechanism.

## Results

### The Tca2 retrotransposon is widespread in *Candida albicans *and polymorphic in nature

**Figure 1:**
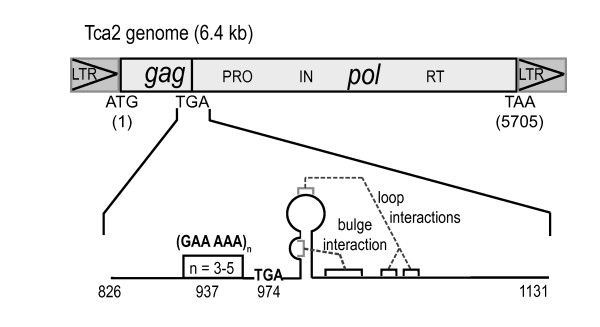
**The Tca2 genome structure**. The Tca2 retrotransposon comprises *gag *and *pol *genes. Within *pol *are arranged the protease, integrase and reverse transcriptase/RNAse H activities (PRO, IN and RT respectively). *Gag *and *pol *ORFs, both in the zero translation frame, are separated by a TGA stop codon. The positions of the potential pseudoknot and polymorphic repeat are shown (see text for details).

The sequence of the Tca2 retrotransposons from *C. albicans *strains hOG1042 [17] and SC5314 [30] revealed that the *gag *TGA stop codon is followed by sequence capable of forming an mRNA pseudoknot structure (Figure 1). In MuLV, a similar pseudoknot directs *gag *stop codon readthrough [21]. To assess the importance or otherwise of the Tca2 pseudoknot, the degree of sequence conservation within 150 nucleotides either side of the *gag *stop codon was assessed. *Gag-pol *junction regions of Tca2 retroelements from a diverse array of eight clinical *C. albicans *isolates from different parts of the world were sequenced and compared.

This analysis revealed that the Tca2 sequences were present in all eight strains tested, and that pseudoknot sequence downstream of the *gag *TGA codon was completely conserved at the nucleotide level within the strains surveyed (data not shown). However, five codons before the stop codon, a GAA AAA repeat motif was discovered (encoding glutamate-lysine, or EK, repeats; see Figure 1), with a polymorphic copy number (n = 3–5), dependent upon strain. This polymorphism 5' of the stop codon might be capable of modulating *gag *UGA recognition, because some nascent polypeptide sequences can influence translation termination efficiency [31]. Overall, the complete conservation of the pseudoknot sequence at the nucleotide level suggests this potential RNA structure may be functional.

### The Tca2 *gag-pol *junction region directs significant expression of a downstream cistron

The strong organisational similarity of Tca2 and the MuLV stop codon contexts suggests that *cis *mRNA sequences surrounding the Tca2 *gag *stop codon direct translational readthrough to achieve *pol *expression [17]. Translational recoding *cis*-signals that direct frameshifting or stop codon readthrough frequently function in translation systems of divergent species. For example, the Tobacco Mosaic Virus stop codon readthrough context also functions in yeast and human as well as the natural plant host [32]. The HIV -1 frameshift signal functions in yeast and *E. coli *as well as the natural human host [32,33]. Accordingly, the Tca2 stop codon region was tested for its ability to direct translational stop codon readthrough in the related yeast *S. cerevisiae*, in which a well-established dual reporter *lacZ-luc *vector system has been used to characterise translational recoding signals from a number of species [32]. By cloning the Tca2 *gag-pol *junction region in-frame between the translationally fused *lacZ *and *luc *genes, *gag *stop codon readthrough frequency could be assessed by measuring luciferase expression relative to that of *lacZ*. On the basis of reported retroelement *gag-pol *stoichiometry, luciferase expression levels in the *gag *TGA stop codon construct would be expected to be 5% of those in a *gag *TGT sense codon control.

**Figure 2:**
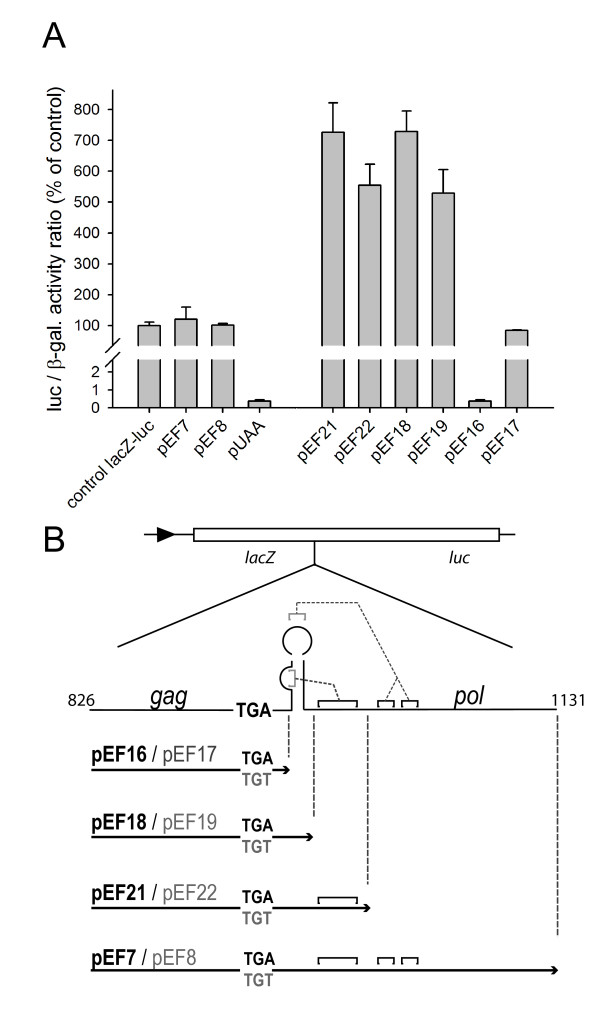
**The influence of the *gag-pol *junction region in directing *pol *expression**. The Tca2 *gag-pol *junction region was cloned in between translationally-fused *lacZ *and *luc *genes in the dual reporter vector pAC98-U. Panel A: the graph shows the normalised level of downstream *luc *gene expression measured in cells transformed with the vectors indicated in the 3' deletion series (see panel B). Luciferase expression was first calculated as the ratio of luciferase to β-galactosidase levels, which was then expressed as a percentage of the same ratio measured using the parental vector (pAC98-U; control *lacZ*-*luc*). Bars represent means of independent transformants +/- 1 standard deviation (n = 3). Constructs tested all contain 5xEK repeats upstream of the *gag *stop codon (see Figure 1).

In fact, the Tca2 *gag-pol *junction region did allow expression of the downstream *luc *reporter, but to a far greater extent than anticipated. Luciferase expression levels measured using the dual reporter vector containing cloned wild-type Tca2 sequences (with 5EK repeats) were 120% of those measured with a control vector in which the TGA *gag *stop codon had been replaced with a TGT sense codon (pEF7 and pEF8; Figure 2). Similar results were obtained for other cloned *gag-pol *junctions containing three or four EK repeats (data not shown). In contrast, inserting a control TAA stop codon between the two reporter genes generated the expected 0.3% readthrough, typical of ordinary stop codons with low level 'leakiness' (pUAA; Figure 2).

To determine if the conserved pseudoknot-containing sequences of the *gag-pol *junction region were important for this stimulation of downstream reporter expression, a 3' deletion analysis was performed on the *gag-pol *junction region (Figure 2B). The results revealed that 3' deletions of pseudoknot sequence further increased gene expression stimulatory activity (pEF21 and pEF22; Figure 2), indicating these sequences must exert some form of repressive effect on the ability of this region to stimulate downstream gene expression. When the deletion was extended to remove all secondary structure, leaving only the purine rich 8 nt immediately downstream of the stop codon, this activity was totally abolished, leaving only 0.3% apparent stop codon readthrough (pEF16; Figure 2).

### The *gag-pol *junction sequence directs *de novo *pol translation initiation events

**Figure 3:**
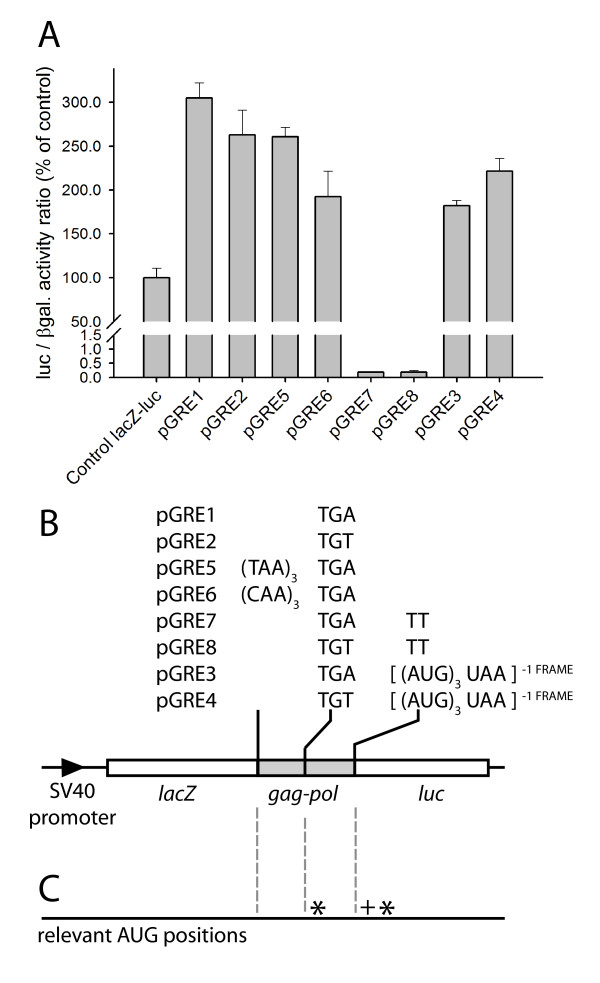
**Expression of *pol *directed by the isolated *gag-pol *junction region is predominantly independent of *gag *translation**. The Tca2 *gag-pol *junction region was cloned in between translationally-fused *lacZ *and *luc *genes in the dual reporter vector pAC98 (Materials and Methods). *Panel A*: the bar chart shows the normalised level of downstream *luc *gene expression in constructs transformed with the parental vector pAC98 (control *lacZ-luc*), wild-type Tca2 stop and sense variants (pGRE1 and 2), junction variants with three in-frame stop codons or three CAA (Gln) codons, respectively, cloned at the 3' end of *lacZ *(pGRE5 and 6), junction variants with the *luc *ORF placed in the -1 frame with respect to *lacZ *(pGRE 7 and 8), and junction variants with three -1 frame AUG codons followed immediately by a -1 frame stop codon, introduced at the 5' end of the *luc *ORF (pGRE 3 and 4). Constructs are depicted schematically in panel B, and panel C indicates the positions of relevant natural AUG codons found in the 0 (*) and +1 frames (+) in the wildtype, non-mutagenised, Tca2 junction region (see text for details). Bars represent means normalised luciferase activities of independent transformants +/- 1 standard deviation (n = 3).

The discovery that the *gag-pol *junction region can direct levels of luciferase expression of up to 400% of a control construct is inconsistent with a simple model of stop codon readthrough, where only a maximum level of 100% of control luciferase expression is possible. To investigate this further, any contribution to luciferase expression from stop codon readthrough at the *gag *UGA stop codon was eliminated by the insertion of three in-frame TAA stop codons at the 3' end of *lacZ*, 190 bases upstream from the native *gag *stop codon, generating plasmid pGRE5 (Figure 3B). The stop codons were placed in an optimal context for release factor recognition [34], and they isolated the Tca2 and luciferase sequences from any ribosomes translating the *lacZ *open reading frame. The corresponding control comprised a TGA stop codon at the junction of *gag *and *pol*, in addition to three CAA Gln codons inserted at the 3' end of *lacZ *(pGRE6). However, introducing extra stop codons at the end of *lacZ *in pGRE5 left the level of luciferase expression unchanged, and comparable to the amount of luciferase produced by the control plasmid pGRE6, or by the progenitor parent construct lacking the triple stop (pGRE1; Figure 3A). In each case, the amount of luciferase production was approximately 250% of that produced by the parent pAC98 plasmid, in which *lacZ *was translationally fused to *luc *with no intervening Tca2 sequence (Figure 3; control *lacZ-luc*).

The ineffectiveness of the three stop codons in pGRE5 at eliminating luciferase expression indicates that downstream, *de novo *translation initiation events must be driving luciferase expression. Only two 0-frame AUG codons are found in the sequence immediately downstream of *lacZ *(Figure 3C), one of which must act as the point of translation initiation for *luc *ORF expression. The first (5'-most) of these AUGs lies in the loop of the pseudoknot stem in the *pol *sequence, with the second lying 18 nucleotides into the *luc *ORF (Figure 3B). To identify which of these two AUG codons was being used to initiate *luc *ORF translation, a -1 frameshift mutation was introduced in between the two AUG codons, at the junction of Tca2 segment and the *luc *ORF. The -1 frameshift mutation would prevent luciferase expression if translation were initiating at the first 0-frame AUG upstream of the frameshift mutation position, but would be silent if the second 0-frame AUG codon were being used downstream of the frameshift position. In fact the -1 frameshift mutation totally abolished luciferase expression, indicating the AUG in the zero frame, at the top of the pseudoknot loop, was acting as a site of *de novo *translation initiation (pGRE7 and 8; Figure 3).

To confirm this, a short ORF in the minus-1 frame was inserted downstream of the first 0-frame AUG codon, at the junction of Tca2 segment and the *luc *ORF. This comprised three minus-1 frame AUG codons followed immediately by a -1 frame stop codon (pGRE3 and a *gag *TGT sense codon counterpart pGRE4; Figure 3B). If translation were initiating at the first 0-frame AUG, the downstream block of three -1 frame AUGs in pGRE5 would not prevent luciferase expression; this was in fact the case (Figure 3A). It seemed likely that this novel translation initiation event at the apex of the pseudoknot loop was occurring either via an efficient internal ribosome entry segment (IRES), allowing ribosomes to bind internally to a *gag-pol *mRNA to translate *pol*, or alternatively because the Tca2 *gag-pol *junction region contains a cryptic promoter element driving expression of a *pol*-only mRNA.

### *Gag-pol *junction sequence contains a novel promoter activity

**Figure 4:**
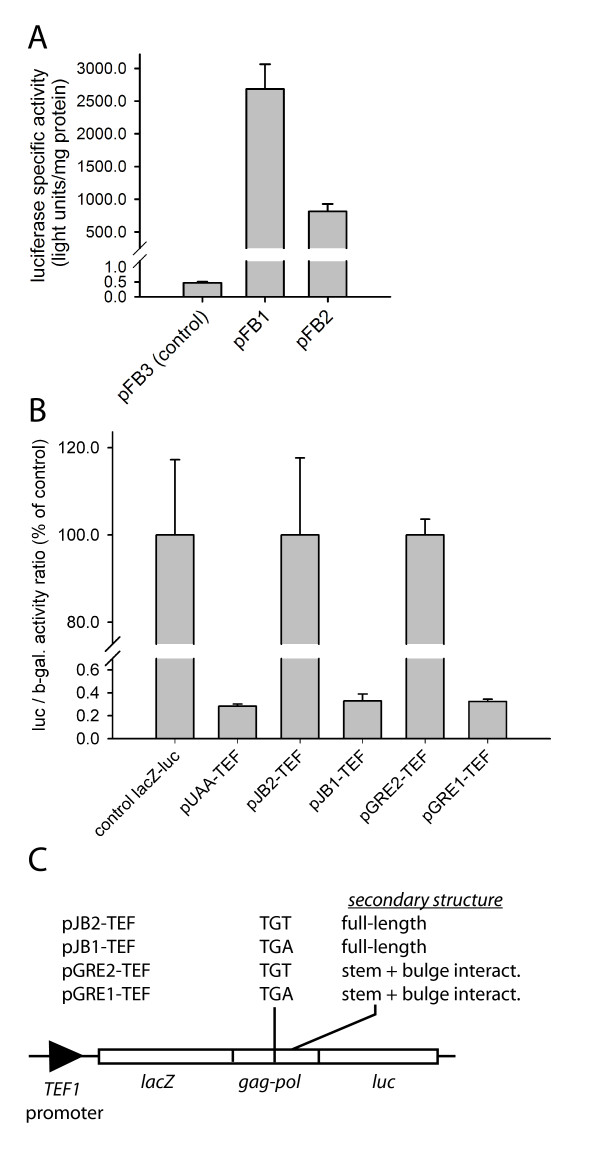
**The Tca2 *gag-pol *junction region contains a novel promoter activity**. The SV40 promoter that directs expression of the *lacZ-luc *translational fusion in the pAC98 vector was deleted in a series of Tca2 *gag-pol *junction constructs to determine if the Tca2 *gag-pol *junction region contained a promoter activity. *Panel A *portrays the specific luciferase expression level in cells transformed with (i) the control parental vector pFB3 lacking an SV40 promoter and any Tca2 sequence (ii) SV40 promoter deletion constructs containing stop (TGA) and sense (TGT) variants of the Tca2 junction region (pFB1 and pFB2 respectively). The SV40 promoter that directs expression of the *lacZ-luc *translational fusion in the pAC98 vector was replaced with the strong *S. cerevisiae TEF1 *promoter to determine the extent of any *gag *stop codon readthrough. *Panel B*: the bar chart shows the normalised level of downstream *luc *gene expression in cells transformed with (i) the parental vector pAC98-TEF (control *lacZ-luc*) (ii) a construct containing a TAA stop codon in a good termination context at the 3' end of the *lacZ *ORF (pUAA-TEF) (iii) constructs containing stop (TGA; pJB1-TEF) and sense (TGT; pJB2-TEF) variants of the full-length Tca2 junction region (nt. 826–1131; Figure 1) (iv) a construct containing stop (TGA; pGRE1-TEF) and sense (TGT; pGRE2-TEF) variants of the Tca2 junction region with a partial 3' deletion. Bars represent means of independent transformants +/- 1 standard deviation (n = 3). Constructs are depicted schematically in panel C.

To test whether downstream cistron expression was being driven by a separate promoter in the Tca2 junction region, the SV40 promoter that directs *lacZ-luc *fusion expression, and most of the *lacZ *ORF, was removed from dual reporter constructs containing (i) the wild-type Tca2 sequence, thus generating pFB1, (ii) the mutated Tca2 in which the *gag *stop codon was changed to TGT, generating pFB2, and (iii) the parental pAC98 control constructs (encoding the *lacZ-luc *fusion), generating pFB3. Any luciferase expression detected in cells transformed with these plasmids must be driven by the putative promoter activity in the Tca2 region. The results show clearly that the control vector pFB3 (lacking any Tca2 sequences) directed only trace luciferase expression, ruling out the possibility that the engineered vector itself contained any significant promoter activity 5' of the *luc *gene (Figure 4A). However, both vectors containing Tca2 junction sequence upstream of the *luc *gene were capable of driving levels of luciferase expression approximately 5000-fold greater than the control vector pFB3 (Figure 4A), indicating that the Tca2 sequences contain a promoter element functional in *S. cerevisiae*.

The identification of the novel junction promoter activity allowed results of the 3' *gag*-*pol *truncation series to be more fully explained (pEF16; Figure 2). Here, deletion of the stem loop sequences virtually eliminated the ability of the promoter to direct luciferase expression (Figure 2). However, deleting the stem loop also deletes the AUG codon likely to be used for translation initiation on the junction promoter mRNA (Figure 3), requiring initiating ribosomes to scan downstream for the next AUG codon, which in fact lies in the +1 frame with respect to the *luc *(Figure 3C). Ribosomes thus initiate translation in the wrong frame, and luciferase expression is eliminated.

### *Gag-pol *junction flanking regions do not direct stop codon readthrough in *S. cerevisiae*

The preceding experiments strongly indicate the existence of a promoter element in the Tca2 *gag-pol *junction region. However, they do not exclude the possibility that luciferase expression (and in Tca2 itself, *pol *expression) is being driven by composite effects, for example by a combination of stop codon readthrough and by the identified promoter. To examine these possibilities, the weak SV40 promoter that directs expression of the dicistronic *lacZ-luc *constructs in pAC98 [32] was replaced by the strong, constitutive yeast *TEF1 *promoter in a new series of plasmids (Figure 4C). This replacement was intended to drive expression of high levels of *lacZ-luc *mRNA and effectively mask the relatively weak, Tca2-derived, internal promoter activity directing low level expression of *luc*-only mRNA.

As expected, the new *TEF1 *promoter vectors produced 1000-fold greater specific-β-galactosidase activity compared to the original pAC98-based vectors (data not shown). When a control stop codon was cloned in between *lacZ *and *luc *genes, *luc *expression dropped to 0.3 % of control, again as expected (pUAA; Figure 4B). When the Tca2 *gag-pol *junction sequence containing the *gag *TGA stop codon was tested using the *TEF1 *promoter vector (pJB1-TEF), *luc *expression was also about 0.3% of a control *gag*-TGT construct (pJB2-TEF; Figure 4B, C). This result clearly shows that the *gag-pol *Tca2 junction sequence does not stimulate luciferase expression in *S. cerevisiae via *stop codon readthrough, or an IRES activity, since Tca2-driven luciferase expression was identical to that measured in the control stop codon construct pUAA-TEF. Identical background levels of stop codon readthrough were also measured using additional *gag*-UGA and counterpart sense TGT constructs in which the pseudoknot sequence had been trimmed slightly (pGRE1-TEF and pGRE2-TEF respectively) an independent verification of this important result (Figure 4B, C). The results therefore clearly show that there is no evidence for this 300 nt region of Tca2 being capable of driving stop codon readthrough or IRES initiation in *S. cerevisiae*.

The penultimate codon in the *gag *ORF is a CTG leucine codon. However, because *C. albicans *translates CUG as serine [35], it was important to ensure that expression in *S. cerevisiae *mimicked authentic *Candida *translation, since it is known that the identity of the amino acid encoded by the penultimate codon can affect stop codon recognition [36]. Using site-directed mutagenesis, the Tca2 *gag-pol *junction CTG [Leu] codon was therefore altered to TGC [Ser] so that the *S. cerevisiae *immediate nascent peptide sequence would be identical to that in *C. albicans*. Levels of stop codon bypass of the *gag *TGA codon in these CTG [Leu] – TGC [Ser] mutants were re-assayed using the pAC98-TEF vector system. However, levels of stop codon readthrough remained at background 0.3% levels (data not shown), indicating that translation of the CTG *gag *codon as serine is not a required component of a stop codon readthrough or IRES-type activity.

The results as a whole indicate that a novel, but minor promoter activity in this region was the only element capable of directing expression of a gene downstream of the *gag *TGA stop codon in *S. cerevisiae*, and that additional translational control mechanisms were not operating to drive luciferase expression in Tca2 *gag-pol *constructs. The results also indicate that in contrast to most viral or transposon stop codon readthrough signals, and contrary to expectation [17] the immediate *gag *UGA-flanking sequences in Tca2 (including the putative pseudoknot) are not active in directing readthrough.

### The *gag-pol *junction promoter is functional in *C. albicans*

It was important to determine if the novel promoter activity identified by the experiments in *S. cerevisiae *was also functional in *C. albicans*, and secondly, to determine the efficiency of the *gag-pol *junction promoter relative to that of the long terminal repeat (LTR) promoter that drives transcription of the major *gag-pol *RNA. A transcriptional activity ratio of 1:20 in favour of the LTR promoter might indicate that in this retroelement, *pol *is translated from a *pol*-only mRNA template produced at 5% of the abundance of a *gag-pol *RNA.

To test the junction promoter activity in *Candida albicans*, the Tca2 *gag-pol *junction sequence was cloned upstream of a promoterless *lacZ *reporter in a *C. albicans *integrative plasmid (pCRlacZ). Once integrated into the *Candida *genome, the ability of the Tca2 sequence to act as a promoter and drive *lacZ *activity could be assayed. The pseudoknot AUG codon, identified as the translation initiation point for transcripts driven by the junction promoter (Figure 3), was placed in frame with the *lacZ *sequence. Two vectors were constructed in this way, one carrying the wild-type Tca2 insert (pCRLP-G1), and one with the Tca2 sequence with stop codon mutated to sense (pCRLP-G2). These plasmids were integrated into the *C. albicans *genome and the β-galactosidase specific activity was measured. For comparison, the long terminal repeat (LTR) promoter was also cloned into pCRlacZ, in such a way that the *lacZ *AUG was placed in an equivalent position to that normally occupied by the *gag *AUG, creating pCRLP-LTR.

**Figure 5:**
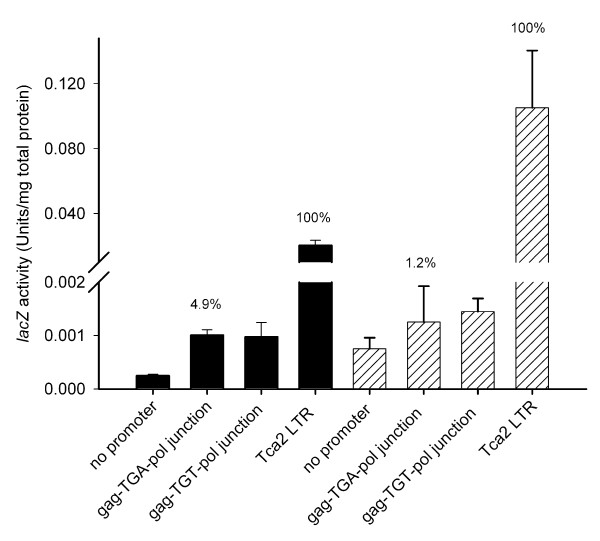
**The Tca2 long terminal repeat promoter, but not the *gag-pol *junction promoter, is heat-shock inducible**. Either the Tca2 LTR promoter, or the newly identified *gag-pol *junction promoter, was cloned upstream of a promoter-less copy of the *S. thermophilus lacZ *gene integrated into the *C. albicans *genome at the *ADE2 *locus. β-galactosidase specific activities were measured in lysates from cultures growing at 30°C (solid bars) or after growth at 23°C followed by a two-hour heat-shock at 37°C (hatched bars). The *gag-pol *junction promoter activity was assayed in this way using either *gag *TGA variant (gag-TGA-pol) or TGT sense codon (gag-TGT-pol). The specific activity of the junction promoter constructs is indicated on the bar chart as a percentage of the activity directed by the LTR promoter (100%). Bars represent the means of three independent cultures. Error bars represent +/- 1 standard deviation.

*C. albicans *integrants for each of the constructs were grown in liquid culture, either at a constant temperature of 30°C, or at a temperature of 23°C, followed by a 2-hour heat-shock at 37°C. The latter condition replicates that under which Tca2 is reported to retrotranspose actively [29]. The results show that at a constant 30°C, the junction promoter has an activity 4.9% of that of the LTR promoter (Figure 5). This activity ratio would produce a ratio of *gag *to *gag-pol *proteins of 20:1, consistent with molar ratios required in other retroelements to generate active retroviruses, although there is no evidence that Tca2 is active at 30°C. Under heat shock conditions, transcription from the LTR promoter increased five-fold, consistent with the reported increase in retrotransposition under these conditions (Figure 5). However, the junction promoter activity was unaffected by heat-shock, meaning that under conditions where the retrotransposon is reportedly most active, the activity of the junction promoter was only 1.2% of that of the LTR promoter. This departs significantly from *gag-pol *ratios reportedly required to produce active retrotransposition. These results therefore challenged the hypothesis that retrotransposition is dependent upon the newly identified junction promoter driving expression of a *pol*-only mRNA template from which Tca2 *pol *proteins are translated, and instead favour the hypothesis that the LTR promoter somehow directs *pol *synthesis.

### The induction properties of the Tca2 *pol *protein match those of the LTR promoter

The discovery that unlike the LTR promoter, the *gag-pol *junction promoter is not heat shock-inducible, makes it unlikely that the junction promoter directs significant *pol *expression during active retrotransposition. Accordingly, evidence was sought that in *C. albicans*, *pol *protein expression is driven from the LTR promoter. Were this to be the case, it would be expected that *pol *protein expression would be significantly induced following a 23°C–37°C heat shock.

**Figure 6:**
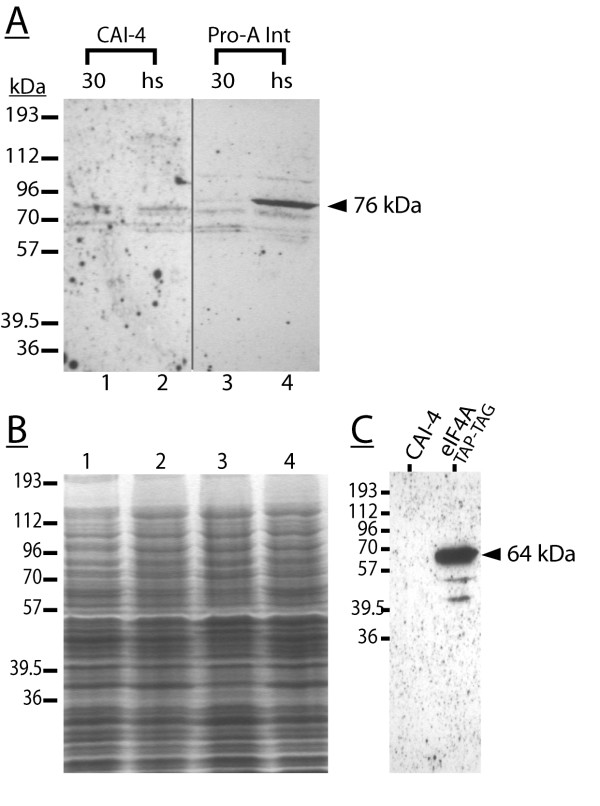
**Tca2 *pol *protein expression is directed by LTR promoter activity in *C. albicans***. The *pol *ORF of a genomic copy of *C. albicans *Tca2 was tagged at its 3' end immediately before the *pol *stop codon with Protein A coding sequence. Panel A; proteins from the untransformed host strain (CAI-4) and a *pol*-Protein A integrant (ProA-Int) were separated (SDS-PAGE), western-blotted, and blots probed with anti-Protein A antibody. Arrowhead indicates the migration position of a 76 kDa protein induced under heat shock conditions, present in the integrant but not the parental strain CAI-4. Results are typical of at least three experiments involving independent Protein A integrant isolates. Panel B; a replicate gel was Coomassie Blue-stained to show equivalent lane loadings. Panel C; the anti-Protein A antibody specificity was confirmed by probing a control blot of untransformed CAI-4 lysate and that from an *S. cerevisiae *strain expressing TAP-tagged eIF4A translation factor [59]. The 65 kDa protein detected by the antibody (indicated by arrowhead) represents eIF4A (44.6 kDa) fused to the TAP tag (20 kDa), the latter containing Protein A.

To test this hypothesis, homologous recombination was used to epitope tag a *C. albicans *genomic copy of Tca2 at the 3' end of *pol*, immediately before the *pol *stop codon, with Protein A coding sequence. Replicate integrants of this type, together with the parental wild-type strain, were grown in liquid culture either at a constant temperature of 30°C, or at a temperature of 23°C, followed by a 2 hour heat-shock at 37°C. Lysates were prepared from these cultures, proteins resolved using SDS-PAGE, western blotted, and blots probed with anti-Protein A antibody. Care was taken to ensure equivalent lane loadings (Figure 6B), that the antibody was specific for Protein A, and that it did not significantly cross-react with any wild-type *C. albicans *proteins (Figure 6C).

The resulting autoradiographs show that in the Protein A-integrant, but not in the parental strain, a protein of 76 kDa that was almost undetectable in the 30°C lysates was induced strongly following heat shock (Figure 6A). This result was reproduced in at least four independently isolated integrants (data not shown). Subtraction of the mass of the Protein A tag (12 kDa) from that of the heat-shock induced protein (76 kDa), leaves a native untagged mass of 64 kDa. This is consistent with an approximate size range for the *pol *reverse transcriptase (RT) protein, the most 3' of the Tca2 *pol *enzyme activities. In fact, *S. cerevisiae *Ty5 retrotransposon RT was measured at 59 kDa using a similar tagging protocol [37]. It was not possible to immuno-detect any putative *gag-pol *fusion protein, since it is expected this would be cleaved by the integral retrotransposon protease activity within *pol*. Crucially, the detection of a Tca2 putative ProteinA-tagged reverse transcriptase that was strongly heat-shock induced directly suggests that *pol *proteins are not expressed from a *pol *RNA-only product of the junction promoter, but in fact are translated from a *gag-pol *mRNA transcribed from the heat-shock inducible LTR promoter. This in turn would indicate that to achieve this translation, in some way the translation terminating effect of the *gag *stop codon within a *gag-pol *transcript must be bypassed.

## Discussion

Translational control mechanisms are frequently employed to achieve regulated *gag-pol *expression in different retroelements. Many examples are known of viral or retrotransposon stop codons that are readthrough at a given frequency during translation, allowing controlled expression of downstream coding information [20,24-26]. In Tca2, the *gag *UGA stop codon, sited upstream of a potential RNA pseudoknot sequence, was suggested to be an obvious candidate for this type of programmed translational readthrough. The nucleotide environment of the Tca2 *gag *stop codon, which includes a downstream potential RNA pseudoknot sequence, was very similar to that of the MuLV *gag *stop codon, which is readthrough at a frequency of 5% to allow *pol *expression [20]. However, in this work, experiments in *S. cerevisiae *testing the hypothesis using dicistronic constructs produced some surprising results. The measured 'readthrough' frequencies in excess of 100% were a clear indication that the Tca2 sequence must direct at least a proportion of luciferase expression *via *mechanisms other than stop codon readthrough (Figure 2), and in fact a minor promoter activity was found within the junction sequence that explained this (Figure 4A). Although attempts to detect this mRNA using Northern blotting were ultimately unsuccessful because of the very low junction promoter activity (data not shown), the *gag-pol *junction sequence was able to drive expression of a promoter-less reporter in *C. albicans*, indicating it did have limited promoter function in both *S. cerevisiae *and its natural host, albeit minor (Figure 5). Analysis of this region identified neither known transcription factor binding sites, nor a TATA box element (data not shown) reinforcing the view that this is a weak promoter. However, it cannot be excluded that this junction promoter plays some role in Tca2 retrotransposition, although clearly this would require a mechanism to recruit free *pol *proteins into the Tca2 capsid. Moreover, since this novel promoter was not induced by heat-shock, conditions that are known to greatly increase the LTR promoter activity, *pol *protein expression and the frequency of Tca2 retrotransposition (Figure 5, Figure 6) [29], it is apparent that the LTR promoter, rather than the junction promoter, plays the dominant role in directing *pol *expression.

The identification of a novel *gag-pol *junction promoter activity did not exclude the formal possibility that the measured 'readthrough' levels of 120% in *S. cerevisiae *were perhaps a composite of 5% stop codon readthrough and 115% of junction promoter activity. For this reason, promoter replacement strategies were employed in *S. cerevisiae*, switching the very weak SV40 promoter that normally drives the *lacZ-luc *constructs with the powerful constitutive *TEF1 *promoter from the yeast translation elongation factor EF1-α. This had the effect of masking the low numbers of transcripts produced by the junction promoter. Using this approach, readthrough levels of 0.3% of control were measured, no different from those of an ordinary, control, stop codon (pUAA; Figure 4B). Based on this evidence, we concluded that this 300-nucleotide region of Tca2 was incapable of directing translational stop codon readthrough, at least in *S. cerevisiae*. This result, although negative, is important. Viral *cis *RNA sequences that flank stop codons and trigger translational readthrough have two key properties; (i) they are limited in their extent to the immediate stop codon environment, often comprising just 6 nucleotides either side of the termination codon and (ii) they are frequently 'portable', that is to say they are functional in non-host systems. The TMV stop codon readthrough signal functions in *S. cerevisiae *and human, for instance [32], and in *Xenopus *[38]. The fact that Tca2 UGA ribosomal readthrough was undetectable in *S. cerevisiae *indicates strongly that the Tca2 sequence comprising 300 nt centred on the *gag *stop codon does not represent a (complete) stop codon readthrough signal. Tca2 *pol *expression does not therefore conform to the TMV or MuLV readthrough paradigms.

Despite the lack of detectable *gag *UGA readthrough activity in *S. cerevisiae*, epitope tagging of the reverse transcriptase sequence of genomic Tca2 in *C. albicans *showed unequivocally that the expression pattern of the tagged *pol *protein was identical to the heat-shock transcriptional responses of the LTR promoter, but not those of the *gag-pol *junction promoter (Figures 5 and 6). This clearly indicates that in *C. albicans*, *pol *proteins are translated from the *gag-pol *RNA required for genome replication and transposition. This in turn infers that the in-frame UGA stop codon must be bypassed in some way in *C. albicans*. What mechanisms could explain such bypass? We consider two explanations possible. The first is that the UGA stop codon is inefficiently spliced from the *gag-pol *RNA to produce, at low level, an mRNA encoding a *gag-pol *translational fusion. Regulated splicing of a *gag-pol *RNA to allow *gag*-independent production of *pol *proteins occurs in foamy viruses, a type of retrovirus [39-42]. However, since *S. cerevisiae *and *C. albicans *splice signals are highly similar [43], it would be expected that 'stop codon splicing' would have been replicated in the *S. cerevisiae *dual reporter experiments, and we therefore consider this explanation unlikely. The second explanation is that despite the finding in *S. cerevisiae *that Tca2 does not conform to the TMV readthrough signal model, Tca2 does achieve *pol *expression *via *translational readthrough, but that this is dependent on additional *cis *sequences remote from the large 300 nt window tested. This is the case in Barley Yellow Dwarf Virus, where sequences up to 750 nt. 3' of the stop codon are required for readthrough [44]. This possibility was not addressed by our survey of 300 nt. surrounding the *gag *stop codon in *S. cerevisiae *because incorporating large sequence windows into the dicistronic reporter construct may have interfered with protein stability (Buchan, J.R. Stansfield, I, unpublished), but future investigations could address this possibility.

It is finally possible that a *Candida*-specific *trans *factor might be required to direct stop codon readthrough, explaining why readthrough was not detectable in the *S. cerevisiae *test-bed system. However, the similarity of the *C. albicans *translational apparatus to that of *S. cerevisiae *favours the expectation that Tca2 stop codon readthrough should be functional in baker's yeast. In plant translation systems, programmed stop codon readthrough of viral UGA stop codons is driven in different cases by one of either cysteinyl tRNA_GCA_, tryptophanylt RNA_CCA_, or arginyl tRNA_UCG _([45-47]. *S. cerevisiae *and *C. albicans *cysteinyl tRNAs have identical anticodon loop sequences, and neither species has an arginyl tRNA_UCG _in their genome. The *S. cerevisiae *tryptophanyl tRNA, with a 2'-O-methylcytidine at position 32 in the anticodon loop [48], does differ from its *C. albicans *counterpart at this position (U32), and this could in theory differentiate the decoding properties of the two tRNAs. However, the *S. cerevisiae *tryptophanyl tRNA is a known functional UGA suppressor [49], and therefore *S. cerevisiae *tRNA^Trp ^should be capable of mediating Tca2 *gag *UGA readthrough. However most important in this discussion is the repeated observation that mRNA *cis *factors, rather than *trans *factors, are centrally important in stop codon readthrough signals, and where tested, these have proved functional in non-host translation systems e.g. [32]. The expectation is therefore that if stop codon readthrough were being driven by the 300 nucleotide window of *gag-pol *junction *cis *sequences in *C. albicans*, readthrough at some level would have been detected in the *S. cerevisiae *dicistronic constructs.

## Conclusion

Our work has demonstrated firstly that a novel Tca2 promoter activity lies at the junction of *gag *and *pol*. Although the evidence does not indicate a central role for this promoter in bulk *gag-pol *synthesis, in *C. albicans *this novel promoter may contribute subtly to Tca2 regulation under non-induced conditions. Secondly, this study has unequivocally demonstrated that counter to predictions, local *cis *mRNA sequences surrounding the Tca2 *gag *stop codon, including a putative pseudoknot, are insufficient to direct stop codon readthrough in baker's yeast, although *gag *stop codon readthrough was not assayed in *C. albicans*. No evidence to support the existence of the pseudoknot was obtained from this study. The Tca2 *gag *stop codon context does not therefore confer termination 'leakiness' as those of MuLV *gag *or the TMV replicase do. It is therefore possible that sequences directing readthrough are multiple, remote and scattered over the Tca2 element, rather than simply stop codon-flanking. Finally, evidence is presented that in *C. albicans*, *pol *proteins are heat-shock induced, as is the activity of the LTR promoter, indicating that in this organism*, pol *proteins are translated from a *gag-pol *RNA using a novel mechanism for stop codon bypass. The study overall prompts a reexamination of the default explanation that immediate stop codon contexts direct translation termination readthrough in viral and transposon systems.

## Methods

### Microbial strains used and growth conditions

*Saccharomyces cerevisiae *strain BY4742 (*MAT ura3Δ0 leu2Δ0 his3Δ1 lys2Δ0*) was used throughout this work. For all propagation of cloned DNA, *Escherichia coli *strain XL1-Blue (*recA1 endA1 gyrA96 thi-1 hsdR17 supE44 relA1 lac *[F *proAB lacI*q *Z*Δ*M15 *Tn*10 *(Tet)]; Stratagene) was used. *Candida albicans *strains used as sources of template DNA for Tca2 retrotransposon PCR amplification were as follows (geographical origin); F37 (UK), TW3-55 (USA), F33 (UK), L485 (UK), S3 (Italy), B59630 (Germany), J990102 (Belgium), SC5314 (genome sequenced strain) [50]. Genomic integrations of reporter constructs into *C. albicans *were carried out using strains CAI-4 (*ura3:: imm434/ura3:: imm434*) or CAI-8 (*ura3:: imm434*/*ura3:: imm434, ade2::hisG*/*ade2::hisG *; [50], depending upon the selectable marker to be used. Yeast growth conditions were as described [50].

### Plasmids constructions and recombinant DNA methods

Standard methods were used for all DNA manipulations [51]. Oligonucleotides used are listed in Table 1. To screen Tca2 *gag-pol *junction sequences, a 304 nt fragments of DNA centred on the *gag *UGA stop codon, were amplified using *Pfu *DNA polymerase and primers ol-364 and ol-365, using as template *C. albicans *genomic DNA preparations from different strains. Amplified products (3'A-tagged) were subcloned into pGEM T-easy (Promega). Clones were sequenced on both strands. Multiple clones were sequenced from each strain.

**Table 1:** Oligonucleotides used in this study

**Primer name**	**Sequence (5'-3')**
ol-364	CCA GAG GAG AAC TCA CTG G
ol-365	CAC AAC AGA TAT TGT GGC ACC
ol-385	GCT AGA GCG GCC GCC AGA GGA GAA CTC ACT GG
ol-386	ACG GTG GCG GCC GCA CAA CAG ATA TTG TGG CAC C
ol-398	GAA AAA CTT CAC TGG AAT GTA AAA CAG GTG CTG CTT C
ol-399	GAA GCA GCA CCT GTT TTA CAT TCC AGT GAA GTT TTT C
ol-420	ATG CGT ATA GCT AGC GGC CGC CCT GTT TTT CAT TCC AGT GAA G
ol-421	ATG CGT ATA GCT AGC GGC CGC CCT GTT TTA CAT TCC AGT GAA G
ol-422	ATG CGT ATA GCT AGC GGC CGC GGA AAC GTG GTT TTG CTG C
ol-424	ATG CGT ATA GCT AGC GGC CGC TTT TCT ACT GGA AAC GTG GTT TTG C
ol-467	CAT ATC GCT AGT AGC GGC CGC TAT AAG GGT AAG GGT AAG CAG AGG AGA ACT CAC TGG
ol-468	CAT ATC GCT AGT AGC GGC CGC TAC AAG GGC AAG GGC AAG CAG AGG AGA ACT CAC TGG
ol-466	CAT ATC GCT AGT AGC GGC CGC CTT ACC CCA TGT TCA TTT TCA TTC AGA GAA TGA GAA TTA AGA TAA GG
ol-545	GAA AAA ACT TCA TCG GAA TGA AAA ACA GGT GCT GCT TC
ol-546	GAA GCA GCA CCT GTT TTT CAT TCC GAT GAA GTT TTT TC
ol-547	GAA AAA ACT TCA TCG GAA TGT AAA ACA GGT GCT GCT TC
ol-548	GAA GCA GCA CCT GTT TTA CAT TCC GAT GAA GTT TTT TC
ol-491	GGT GCT AGC GTC TTC CAT TTT ACC AAC AGT ACC GGA ATG CTG TTC TCG AGT TTT GTG TTC TTA AG
ol-492	TTG TGT TAC ATT CTT GAA TGT CGC TCG CAG TGA CAT TAG CTG CAA GGT GAC GGA TCG ATC
ol-499	ATG CGT ATA GCT ACT CGA GTT TTC TAC TGG AAA CGT GGT TTT GC
ol-500	ATG CGT ATA GCT AGC GGC CGC TTT CTA CTG GAA ACG TGG TTT TGC
ol-550	ATG CGT ATA GCT ACT CGA GTT TGC GGA ACT CAT CTT GTG
ol-551	TAG CTA TAC GCA TGC GGC CGC GCT GTT GCT GTT GCT
ol-626	CAT ATC GCT AGT ACC CGG GGC ATG TCA CGA GCA AAG AAG
ol-627	CGC TAG TAA TGC ATT TCT GCC GTT ATT GCA TTT TG

### Tca2 *gag-pol *junction region cloning

Different Tca2 *gag-pol *junction regions were PCR amplified using primers ol-385 and ol-386. The *Not*I-digested PCR products were cloned into the dual-reporter vector pAC98-U (*CEN*, *URA3*) ; except for a unique *Not*I cloning site and *URA3 *selectable marker, almost identical to pAC99 [52]. This introduced *gag-pol *sequences in between, and in translational frame with, the *lacZ *and *luc *ORFs, generating plasmid pEF3 (3EK repeats), pEF5 (4EK repeats) and pEF7 (5EK repeats). Cloned EK repeat polymorphic variants in pGEM T-easy were mutagenised using primers ol-398 and ol-399, changing the *gag *TGA stop codon to a TGT cysteine codon, generating pGEM-Tca2-TGT-3EK, -4EK and -5EK. These mutagenised *gag-pol *junction regions were amplified (primers ol-385 and ol-386) and sub-cloned into pAC98-U at the *Not*I site, generating pEF4 (3EK repeats with a TGT sense codon; 3EK-sense), pEF6 (4EK-sense) and pEF8 (5EK-sense).

### Deletion analysis of the *gag-pol *junction region

A 3' deletion series of the Tca2 *gag-pol *junction sequence in pAC98-U was constructed. In each case, *gag *TGA and counterpart control, TGT, sequences were amplified by PCR and cloned into pAC98-U on a *Not*I-ended fragment. A 5EK repeat *gag-pol *junction region containing a pseudoknot with bulge complementary sequence, but no loop complementary sequence was amplified (ol385, ol424) and cloned; plasmids pEF21 [TGA] and pEF22 [TGT] generated. A junction region with a stem loop lacking any loop or bulge downstream complementary sequence was amplified (ol385, ol422), and cloned; plasmids pEF18 (TGA) and pEF19 (TGT) generated. Finally, ol-385 was paired with either ol-420, or ol-421, to amplify respectively either A *gag*-TGA-*pol *or *gag*-TGT-*pol *junction region containing 5EK repeats that retained 8 purine rich nucleotides downstream of the stop codon, but lacked any secondary structural elements was amplified (ol385, ol420, TGA variant, or ol385 with ol421, TGT variant) and cloned; plasmids pEF16 and 17 respectively generated. An identical strategy was used to clone the 5EK repeat *gag-pol *junction region containing a pseudoknot with stem loop and bulge complementary sequence, but no loop complementary sequence, into pAC98 (*CEN, LEU2*) generating pGRE1 (TGA stop codon variant) and pGRE2 (TGT sense codon variant).

### Dual reporter vector promoter engineering

Using *Nde*I digestion and re-circularisation of the remaining vector, the SV40 promoter directing expression of the *lacZ-luc *translational fusion, together with all but the last 103 nt of the *lacZ *ORF, was removed from vectors pEF7, its sense codon counterpart pEF8, and pAC98-U, generating respectively plasmids pFB1, pFB2 and pFB3. Separately, in plasmids pAC98-TEF, pGRE1-TEF, pGRE2-TEF, and pGRE5-TEF, the SV40 promoter that directed expression of *lacZ-luc *in the pAC98 derivative vectors was replaced by the strong *S. cerevisiae TEF1 *promoter using homologous recombination in *S. cerevisiae*. pAC98, pGRE1, pGRE2 and pGRE5 were each gapped with the restriction enzyme *Bsm*I, cutting 6 nucleotides before the *lacZ *ATG, and co-transformed into yeast with a PCR-amplified *TEF1 *promoter fragment (using primers ol-491 and ol-492) with 5' and 3' ends complementary to 40 nucleotides either side of the gap site. The repaired vectors were recovered for propagation and verification in *E. coli*. Plasmids pJB1 and pJB2 pAC98-derivative vectors, with *TEF1 *promoter driving *lacZ-luc *expression and containing a full-length *gag-pol *junction region with all pseudoknot sequences, were also created in a similar way using homologous recombination gap repair.

### Directed mutagenesis of the Tca2 *gag-pol *junction region

Plasmids pGRE5 and pGRE6 consisted of pAC98-derivative vectors carrying cloned *gag-pol *junction sequence with extra in-frame stop or sense codons respectively, introduced at the 3' end of the *lacZ *sequence and prior to *gag-pol *junction sequences. These were generated by PCR amplifying the Tca2 *gag*-TGA-*pol *junction sequence containing 5 EK repeats from pEF21 using either ol-467 and ol-424 (pGRE5), or ol-468 and ol-424 (pGRE6). In vectors pGRE3 and pGRE4, an additional -1 frame small ORF (3× ATG followed by TAA) stop codon, was introduced at the 3' end of the *gag-pol *junction sequence immediately before the start of the *luc *ORF. This was achieved by amplifying (using ol385, ol466) the pEF21 Tca2 *gag-pol *junction sequence, or the corresponding *gag *TGT sense variant in pEF22. The resulting PCR fragment was cut with *Not*I, and cloned into *Not*1-gapped pAC98. The -1 frameshift constructs pGRE7 and pGRE8 (*gag *TGA and TGT variants respectively) were made by PCR amplifying the Tca2 *gag-pol *junction with primers ol385 and ol500, before cloning into pAC98.

The CTG codon (decoded as serine in *C. albicans*) representing the penultimate sense codon prior to the Tca2 *gag *stop codon was mutagenised to a TCG serine codon using the Stratagene Quick-Change site-directed mutagenesis protocol and primers ol-385 and ol-386. Mutagenised sequences were sequenced on both strands to confirm the mutagenic change before subcloning into pAC98-TEF to generate plasmids pGRE-20 (containing the CTG to TCG leucine codon substitution and the *gag *stop codon) and pGRE-21 (containing the CTG to TCG mutation and the *gag *TGA stop to TGT sense codon mutation).

### *C. albicans *promoter analysis

The Tca2 *gag-pol *junction containing either *gag*-UGA or *gag*-TGT was amplified (primers ol-385 and ol-499). The resulting DNA fragment was cut using *Not*I and *Xho*I restriction enzymes, and subcloned upstream of a promoterless *Streptococcus thermophilus lacZ *gene in vector pCRlacZ [53], to generate pCRLP-G1 and pCRLP-G2 (TGA and TGT variants respectively). Plasmids pCRLP-G1 and pCRLP-G2, each carrying the selectable *ADE2 *gene, were gapped with *Bam*HI and transformed into *C. albicans *CAI-8 (*ade2*). The genomic *ade2::hisG *sequence was targeted and transformants selected on SD medium lacking adenine. A similar approach was used to clone the Tca2 LTR sequence as a promoter control element upstream of *lacZ *in the same vector system. Amplification of Tca2 LTR sequence from *C. albicans *SC5314 genomic DNA using primers ol-551 and ol-550, and subcloning into pCR-lacZ (as described for pCRLPG1) generated pCRLP-LTR. This was similarly integrated in *C. albicans *strain CAI-8 using homologous recombination at the *ade2::hisG *locus.

### C-terminal Protein A-tagging of Tca2 *pol*

Tca2 *pol *sequence, comprising nucleotides 2833-4727 of the 4730 nt-long *pol *ORF, and excluding the *pol *stop codon, was amplified (ol-626 and ol-627) from *C. albicans *SC5314 genomic DNA. The amplified DNA was cut with *Xma*I and *Nsi*I, and cloned into vector ClpACT-C-ZZ [54], generating plasmid pSI-Tca2, in which *pol *sequences were cloned as a translational fusion with a C-terminal Protein A epitope tag. pSI-Tca2 was restriction-digested with *Sgr*AI, which cleaves uniquely at nt 3196 of the *pol *sequence, and the linearised vector, also carrying the *URA3 *gene, transformed into *C. albicans *CAI-4 (*ura3*). Transformants were selected on SD agar minus uridine.

### *C. albicans *and *S. cerevisiae *DNA transformation

Plasmids were transformed into *S. cerevisiae *and *C. albicans *using a standard lithium acetate-based protocol [54,55].

### Reporter gene assays in yeast

*S. cerevisiae *strains transformed with dual reporter vectors were subjected to β-galactosidase and luciferase assay using the Tropix Dual-Light assay kit as directed by manufacturer, modified as described [56]. The assay of each transformant β-galactosidase and luciferase activity was conducted in triplicate, and three independent transformants were routinely assayed in this way.

### Assaying heat-shock induction of β-galactosidase activity in *C. albicans*

*C. albicans lacZ*-integrant strains were grown in liquid culture shake flasks in YPD (containing 2 mg ml^-1 ^uridine) at either 30°C overnight for 10 generations, or at 23°C for 10 generations overnight followed by transfer for 2 hours to 37°C (heat shock conditions). Heat shock transfer was achieved by centrifugal harvest, resuspension in pre-warmed 37°C medium, and decanting into pre-warmed shake flasks. Following incubation, cells were harvested, and lysed using 400 mesh glass beads and a Fastprep cell breakage machine (Thermo Savant, Middlesex, UK) in *Candida *lysis buffer (50 mM Tris HCl pH7.5, 150 mM NaCl, 0.5% NP40 and protease inhibitors (Roche Mini protease inhibitor at manufacturer's recommended concentration, 1 mM phenylmethylsulphonylfluoride, 1 mM pepstatin]). β-galactosidase activities were measured at 37°C using the substrate *ortho*-nitrophenolgalactoside using standard enzyme assay procedures, and absorbance monitored (420 nm). Enzyme activities were expressed as specific activities per mg total cell protein.

### Western blot analysis

*C. albicans *cells were lysed in lysis buffer (50 mM Tris pH7.5, 50 mM KCl, 2 mM 2-mercaptoethanol containing protease inhibitors [Roche Mini-protease inhibitor at manufacturer's recommended concentration]) using 400-mesh glass beads (Sigma). Proteins were resolved using 10% acrylamide SDS-PAGE gels [57]. Proteins were semi-dry blotted onto nitrocellulose (Amersham Hybond-C; blotted for 105 minutes at 15 V, 50 mA), and the blot treated with Qentix reagent (Pierce) to improve signal strength. Blots were blocked and probed according to standard protocols [58]. Protein A-tagged proteins were detected using an anti-Protein A mouse monoclonal SPA27 antibody (Sigma) at 1 × 10^-4 ^dilution, and probed with a horseradish peroxidase-conjugated goat anti-mouse secondary antibody, used at 1 × 10^-4 ^dilution. Bound antibodies were detected using Femto-West ECL reagent (Pierce) according to manufacturer's instructions.

## Authors' contributions

EMF established the basic Tca2 clones and the truncation series, and carried out molecular genetic analysis in *S. cerevisiae*. SRN carried out the translation regulation and promoter replacement molecular genetic studies in *S. cerevisiae *and promoter analysis in *C. albicans*. FB carried out the promoter analysis in *S. cerevisiae*. JB participated in truncation series molecular genetic analysis in *S. cerevisiae*. LAG participated in co-ordination and design of the study together with IS. IS conducted the immune analysis, conceived the study and drafted the manuscript. All authors read and approved the final manuscript.
